# The Abundance of Viroid-Like RNA Obelisk-*S.s* in *Streptococcus sanguinis* SK36 May Suffice for Evolutionary Persistence

**DOI:** 10.1007/s00239-025-10250-y

**Published:** 2025-05-09

**Authors:** Rohan Maddamsetti, Lingchong You

**Affiliations:** 1 Department of Biochemistry and Microbiology, Rutgers University, New Brunswick, NJ USA; 2 Center for Quantitative Biodesign, Duke University, Durham, NC USA; 3 Department of Biomedical Engineering, Duke University, Durham, NC USA

**Keywords:** Obelisk, Viroid-like RNA, Evolution, Transcriptomics, Oral microbiome

## Abstract

A new class of viroid-like RNAs, called Obelisks, was recently reported by Zheludev et al*.* (Cell 187:6521–6536.e6518, 2024). They found thousands of Obelisk sequences globally and identified a specific 1137 nt Obelisk, called Obelisk-*S.s*, in monoculture transcriptomes of *Streptococcus sanguinis* SK36, a commensal bacterium of the human oral microbiome. Here, we confirm that Obelisk-*S.s* is highly abundant in SK36, despite its absence from the SK36 genome (i.e., as DNA). In 11 out of 17 monoculture SK36 RNA-seq datasets examined, Obelisk-*S.s* is more abundant than any mRNA. Given its relative abundance, we hypothesized that multiple Obelisk-*S.s* variants could coexist within SK36. We found three Obelisk-*S.s* mutations at 5–10% allele frequency in some samples: a R162R synonymous mutation in one set of replicate transcriptomes, and an I48I synonymous mutation and an intergenic mutation in another set of replicate transcriptomes. A simple mathematical model shows how high Obelisk abundance can transiently stabilize intracellular Obelisk populations, and how extreme Obelisk abundances may stabilize intracellular Obelisk populations indefinitely. Evolution experiments with SK36 could test this theory and could shed light on how Obelisks function and evolve within their microbial hosts.

**Supplementary Information:**

The online version contains supplementary material available at 10.1007/s00239-025-10250-y.

## Introduction

In a recent paper, Zheludev et al. (2024a, b) report the discovery of a new kind of RNA, which they call Obelisks. Obelisks are viroid-like RNAs that are approximately 1000 nt in length, which fold into long hairpins and encode one or two proteins of unknown function. These unknown proteins are called Oblins. Obelisks are found globally and are prevalent in the ocean and in human oral microbiome metatranscriptomes (Zheludev et al. 2024a, b; López-Simón, et al. 2025). Given the novelty of this discovery, we wanted to verify key aspects of this study.

In this report, we use independent transcriptomic data to confirm the existence of Obelisk-*S.s* in *Streptococcus sanguinis* SK36. Importantly, we found no evidence of Obelisk-*S.s* in SK36 genomic data, implying that Obelisk-*S.s* exists as an intracellular population of small viroid-like RNAs within SK36 cells.

In addition, we highlight key aspects of Obelisk biology in SK36, namely its extreme relative abundance, and the presence of low-frequency polymorphisms in Obelisk-*S.s* across RNA-seq technical replicates. These polymorphisms are not conserved across SK36 transcriptomes, suggesting that they arose during SK36 monoculture before cDNA preparation for RNA-seq. The evolutionary dynamics driving these preliminary patterns of Obelisk variation (i.e., mutation rates, strength of selection and distribution of fitness effects, Obelisk population bottlenecks during SK36 cell division and concomitant effects of genetic drift) remain a mystery in need of further investigation. Finally, we built a simple mathematical model to understand Obelisk persistence in SK36. The model shows that sufficiently high Obelisk abundance may stabilize Obelisk populations against loss, even in the absence of any fitness benefit to their host cell, given the assumption that Obelisks are randomly partitioned into daughter cells during cell division.

## Results

### Obelisk-*S.s *is Not Found in the *S. sanguinis* SK36 Genome

We used NCBI BLAST to search for homology between Obelisk*-S.s* and the SK36 genome (Methods). No significant similarity was found. We repeated the search on the entire NCBI Nucleotide collection (nr/nt) database. Again, no significant similarity was found. We asked whether this result was caused by errors in SK36 genome assembly. To do so, we downloaded Illumina resequencing data for SK36 from the NCBI Short Read Archive and jointly mapped reads to the SK36 reference genome found in the NCBI RefSeq database (Accession: NC_009009.1) and the Obelisk-*S.s* reference sequence (Methods). 100% of these sequencing reads mapped to the SK36 reference genome, with 225.7 × sequencing coverage on average. By contrast, no sequencing reads at all mapped to the Obelisk-*S.s* reference sequence. Zheludev et al. (Zheludev et al. 2024a) experimentally confirmed this absence through DNA PCR in monocultures (absent in genomic DNA but detectable via RT-PCR in cDNA). Therefore, our bioinformatic analysis reinforces the previous observation that Obelisk-*S.s* is not found in the *S. sanguinis* SK36 genome.

### Obelisk-*S.s *is Highly Abundant in the *S. sanguinis* SK36 Transcriptome

We then asked whether Obelisk-*S.s* is found in the *S. sanguinis* SK36 transcriptome, as reported by Zheludev et al. (2024a, b). We downloaded 17 RNA-seq runs collected from wildtype SK36 monoculture (Table 1). These data were collected and published by different research groups working independently (Choi, et al. 2018; Helliwell, et al. 2023; Treerat, et al. 2023; Zeng, et al. 2023). Four of these datasets were analyzed by Zheludev et al. (2024a, b). The remaining thirteen datasets were not; these independent data were submitted by researchers at Oregon Health & Science University in 2023 as part of two different projects (Helliwell, et al. 2023; Treerat, et al. 2023). We used *kallisto* (Bray, et al. 2016) (Methods) to map the Illumina reads from these RNA-seq datasets to the SK36 genome and the Obelisk-*S.s* reference sequence (Supplementary Data File 1). Obelisk-*S.s* is highly abundant in all of these samples (Table 1). Since RNA-seq determines relative and not absolute abundances for cellular RNA, we ranked RNA abundance for all 2270 protein-coding genes against Obelisk-*S.s* in each sample. Ranking provides a robust and non-parametric measure of Obelisk-*S.s* abundance in the SK36 transcriptome, allowing us to compare independent RNA-seq datasets collected by different groups using different protocols, without making assumptions about the statistical distribution of RNA counts within or across datasets (Friedman 1937). Obelisk-*S.s* is more abundant than any mRNA in SK36 in 11 of the 17 RNA-seq datasets. Obelisk-*S.s* is ranked no lower than 16 out of 2271 sequences in relative abundance in the remaining 6 RNA-seq datasets (Table 1).

**Table 1:** Wildtype *Streptococcus sanguinis* SK36 RNA-seq datasets analyzed for Obelisk-*S.s*

Bioproject^a^	SRA Run ID	Growth condition^b^	RNA-seq replicate^c^	Previously analyzed	RNA abundance rank of Obelisk-*S.s*^d^
PRJNA270301	SRR1713039	BHI media, oxic	1	Yes	1 of 2271
PRJNA862079	SRR20627698	TY media + glucose, oxic	1	Yes	10 of 2271
PRJNA862079	SRR20627697	TY media + glucose, oxic	2	Yes	6 of 2271
PRJNA862079	SRR20627696	TY media + glucose, oxic	3	Yes	6 of 2271
PRJNA961761	SRR24302371	ASS media, oxic	1	No	16 of 2271
PRJNA961761	SRR24302370	ASS media, oxic	2	No	13 of 2271
PRJNA961761	SRR24302369	ASS media, oxic	3	No	13 of 2271
PRJNA937727	SRR23591557	CDM + sucrose, oxic	1	No	1 of 2271
PRJNA937727	SRR23591555	CDM + sucrose, oxic	2	No	1 of 2271
PRJNA937727	SRR23591553	CDM + sucrose, oxic	3	No	1 of 2271
PRJNA937727	SRR23591551	CDM + glucose, oxic	1	No	1 of 2271
PRJNA937727	SRR23591549	CDM + glucose, oxic	2	No	1 of 2271
PRJNA937727	SRR23591547	CDM + glucose, oxic	3	No	1 of 2271
PRJNA937727	SRR23591545	CDM + sucrose, anoxic	2	No	1 of 2271
PRJNA937727	SRR23591543	CDM + sucrose, anoxic	3	No	1 of 2271
PRJNA937727	SRR23591541	CDM + glucose, anoxic	2	No	1 of 2271
PRJNA937727	SRR23591539	CDM + glucose, anoxic	3	No	1 of 2271

^a^PRJNA270301 was deposited in NCBI SRA in 2014 by Kyungpook National University. PRJNA862079 was deposited in 2022 by the University of Florida. PRJNA961761 and PRJNA937727 was deposited in 2023 by Oregon Health & Science University

^b^All samples were grown at 37 °C. *BHI* Brain–Heart-Infusion, *TY* Tryptone-Yeast, *ASS* Artificial Saliva Solution, *CDM* Chemically Defined Media. See references (Choi et al. 2018; Helliwell et al. 2023; Treerat et al. 2023; Zeng et al. 2023) for details

^c^Replicate 1 for the CDM + sucrose, anoxic and CDM + glucose, anoxic conditions was not found in NCBI SRA, and therefore excluded

^d^This comparison excludes rRNA and sRNA species; we only compare the relative abundance of reads mapping to the 2270 protein-coding genes in the SK36 genome (i.e., mRNA) to the relative abundance of reads mapping to Obelisk-*S.s*

To check the robustness of this finding, we used *breseq* to map the Illumina reads from these RNA-seq datasets to the SK36 genome and the Obelisk-*S.s* reference sequence (Table 2). Even though Obelisk-*S.s* (1137 nt) is less than 0.1% of the size of the SK36 genome (2,388,435 nt), at least 1% of all RNA-seq reads map to Obelisk-*S.s* in all samples.

**Table 2:** *breseq* RNA-seq read-mapping statistics

NCBI SRA Run ID	Number of Illumina sequencing reads	Percentage of reads mapped (%)	Percentage of mapped reads to SK36 genome (2,388,435 nt long) (%)	Percentage of mapped reads to Obelisk-*S.s* (1,137 nt long) (%)
SRR1713039	25,004,887	26.5	97.8	2.2
SRR20627698	16,742,304	99.3	98.5	1.5
SRR20627697	58,571,050	99.3	98.3	1.7
SRR20627696	17,894,187	99.3	98.3	1.7
SRR24302371	73,183,993	66.6	98.8	1.2
SRR24302370	76,465,399	70.2	98.8	1.2
SRR24302369	69,170,853	70.8	98.8	1.2
SRR23591557	27,744,841	97.2	95.9	4.1
SRR23591555	27,356,457	97.5	93.7	6.3
SRR23591553	22,125,927	95.2	86.7	13.3
SRR23591551	33,110,925	97.6	96.1	3.9
SRR23591549	24,870,174	96.8	93.9	6.1
SRR23591547	23,875,329	96.3	88.3	11.7
SRR23591545	23,918,852	96.2	96.1	3.9
SRR23591543	25,021,051	96.2	97.6	2.4
SRR23591541	30,609,207	96.7	97.5	2.5
SRR23591539	25,035,624	96.0	96.9	3.1

Together, these analyses suggest that Obelisk-*S.s* is highly abundant in *S. sanguinis* SK36—even though it is not encoded as DNA in the SK36 genome.

### Evidence of Obelisk-*S.s* polymorphism

Although RNA-seq does not provide quantitative estimates of Obelisk*-S.s* copy number, these data suggest that Obelisk*-S.s* exists at very high copy numbers in SK36 cells (although we cannot rule out the possibilities that Obelisk-*S.s* RNAs are highly enriched compared to mRNAs during RNA-seq processing, or that unknown life stages of *Obelisk-S.s* replication might somehow inflate copy number estimates). Given its apparent cellular abundance, we asked whether single SK36 clones might contain diverse Obelisk subpopulations. By running *breseq* in polymorphism mode (Methods), we screened for segregating mutations in Obelisk.*S.s.* To reduce false positives, we only considered mutations occurring above 5% allele frequency. The results are shown in Table 3. A R162R (CGA → CGG) *Oblin-1* mutation was found at 8–11% allele frequency in 2 out of 3 RNA-seq replicates in NCBI Bioproject PRJNA961761. A I48I (ATC → ATA) *Oblin-1* mutation and an intergenic (+ 274) mutation downstream of *Oblin-1* was found in several samples grown in different conditions in NCBI Bioproject PRJNA937727. By comparing Tables 1 and 3, one can see that these two mutations are always found in samples labeled as “Replicate 2” in this Bioproject. Therefore, it is likely that the apparent parallelism across replicates reflects some underlying correlation in how these samples were prepared, and not independent mutational events across replicate SK36 cultures. For instance, it is possible that these Obelisk*-S.s* mutations evolved in the particular colonies or overnight cultures used to inoculate replicate cultures spanning different conditions for RNA isolation.

**Table 3:**
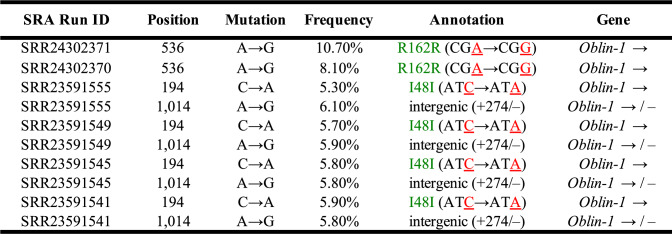
Obelisk-*S.s* polymorphisms found across RNA-seq datasets

*Arrows in the Gene column represent gene orientation in the Obelisk-*S.s* reference sequence. See *breseq* documentation for details on this reporting format for intergenic mutations (https://github.com/barricklab/breseq/wiki/Output)

### A Mathematical Model Shows How High Copy Number Can Stabilize Obelisk Persistence

**Fig. 1:**
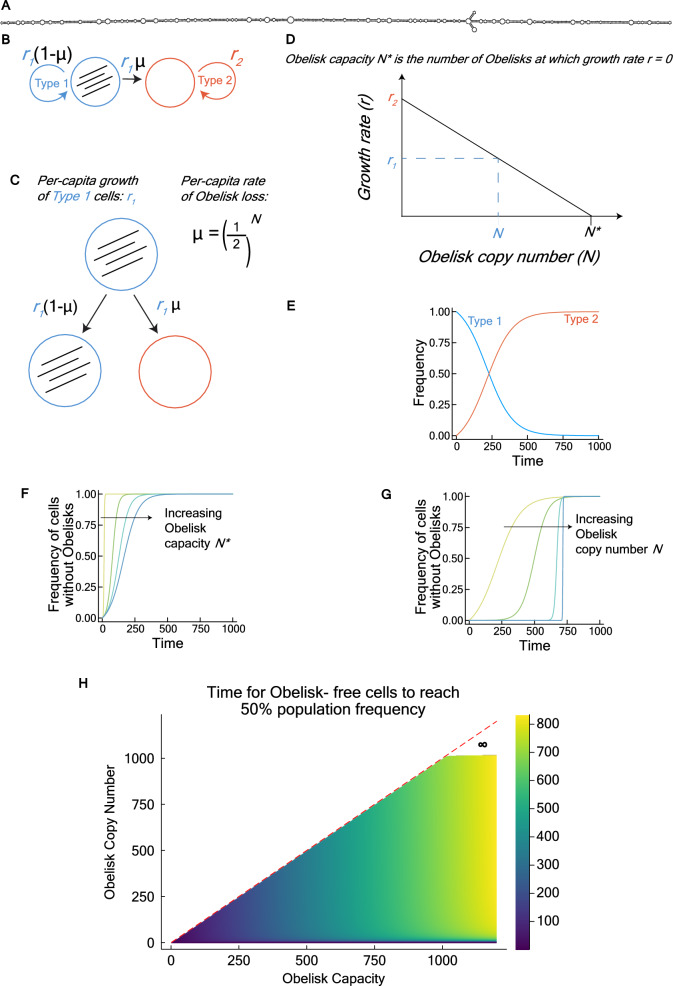
High Obelisk copy number can increase evolutionary stability by reducing the rate of generating Obelisk-free cells. **A** Predicted secondary structure of Obelisk-*S.s.* The structure can be predicted from sequence (provided in the Methods) with *RNAfold* (http://rna.tbi.univie.ac.at//cgi-bin/RNAWebSuite/RNAfold.cgi) and visualized using *forna* (http://rna.tbi.univie.ac.at/forna/). Interested readers can also visualize the mutations at positions 194, 536, and 1,014 using the *forna* tool. **B** State diagram for the mathematical model. The two states represent cells containing intracellular Obelisks (Type 1), and cells that have lost intracellular Obelisks. **C** Type 1 cells grow at rate *r*_1_. A Type 2 cell can be generated from a Type 1 cell at rate *µ* = (1/2)^*N*^, representing the probability of inheriting zero out of N Obelisks in its Type 1 parent due to random Obelisk segregation during cell division. Therefore, Type 2 cells are generated at rate *r*_1_*µ*. **D** Obelisks are assumed to confer a small, linear fitness cost to the host cell. Obelisk capacity N* is defined as number of Obelisks in a cell at which growth rate *r*_1_ = 0. Increasing N* therefore reduces the per-capita fitness burden of an Obelisk. Type 2 cells have zero Obelisks and grow at rate *r*_2_*.* Type 1 cells have *N* obelisks and grow at rate *r*_1_. **E** A typical simulation shows the evolution of Type 2 cells that outcompete Type 1 cells containing Obelisks. The simulation result in this panel uses the following parameter settings: *N* = 10, N* = 1000. **F** Increasing Obelisk capacity N* decreases Obelisk fitness burden and has a small effect on the time for Type 2 cells to establish in the population. Colors shift from yellow to blue as N* increases. *N* is fixed at 10, and N* is varied from 20, 200, 400, 600. **G** As Obelisk copy number *N* increases, Type 2 cells take longer to evolve but establish more rapidly due to a higher Obelisk fitness burden. Colors shift from yellow to blue as *N* increases. N* is fixed at 1000, and *N* is varied from 10, 20, 100, and 800. **H** Increasing Obelisk copy number *N* and Obelisk capacity N* increase the evolutionary stability of Type 1 cells. When *N* is sufficiently high (~ 1100 copies in numerical simulations), the per-capita Obelisk loss rate *µ* = (1/2)^*N*^ goes to zero, and Type 1 cells containing Obelisks become evolutionarily stable

The double-stranded hairpin secondary structure that is predicted to be formed by Obelisk-*S.s* (Fig. 1A) could help protect this RNA from cellular degradation (Patop, et al. 2019). It is easy to see that Obelisks could persist in SK36 if it confers some fitness benefit or if its loss from the cell confers some fitness cost. In addition, its high cellular abundance could explain its persistence in SK36, even in the absence of any selective benefit. We examine the plausibility of this last hypothesis by building a simple mathematical model.

We model a population of Type 1 cells containing Obelisks and Type 2 cells without Obelisks (Fig. 1B). Type 1 cells grow at rate *r*_1_. A fraction *µ* = (1/2)^*N*^ of newly produced Type 1 cells lose the Obelisk due to random segregation at cell division, where *N* is the Obelisk copy number (Fig. 1C). Therefore, Obelisk-free mutants are produced at rate *r*_1_*µ*. Obelisks are assumed to confer a small, linear fitness cost to the host cell. We define Obelisk capacity N* as number of Obelisks in a cell at which growth rate *r*_1_ = 0. Increasing N* therefore reduces the fitness burden of an Obelisk. Type 2 cells without Obelisks grow at rate *r*_2_. Given particular values of the Obelisk copy number *N* and Obelisk capacity N*, it follows that *r*_1_ = *r*_2_ × [1 – (*N*/N*)] (Fig. 1D).

These assumptions lead to the following linear system of ordinary differential equations (Methods):

$$ \frac{{d{\varvec{x}}}}{dt} = \left[ {\begin{array}{*{20}c} {r_{1} \left( {1 - \mu } \right)} & 0 \\ {r_{1} \mu } & {r_{2} } \\ \end{array} } \right]\left[ {\begin{array}{*{20}c} {x_{1} } \\ {x_{2} } \\ \end{array} } \right] $$

where **x** is a vector of two states, *x*_1_ (cells containing Obelisks) and *x*_2_ (cells without Obelisks). An example simulation of this model is shown in Fig. 1E.

Increasing Obelisk capacity N* decreases Obelisk fitness burden and has a small effect on the time for cells without Obelisks to establish in the population, by reducing *r*_1_ (Fig. 1F). Increasing Obelisk copy number *N* increases Obelisk fitness burden but has a larger effect on the time for cells without Obelisks to establish in the population, because the per-capita loss rate *µ* decreases geometrically with *N* (Fig. 1G). By varying *N* and N* and calculating the time for Obelisk-free cells to reach 50% population frequency (Methods), we find that when Obelisks reach copy numbers of ~ 1100 Obelisks per cell, the per-capita Obelisk loss rate *µ* = (1/2)^*N*^ goes to zero (based on a numerical precision limit of (1/2)^1074^ = 5.0 × 10^−324^ and (1/2)^1075^ = 0.0 in our computational notebook), and Type 1 cells containing Obelisks become evolutionarily stable, despite the fitness cost conferred by abundant Obelisks. Such extreme copy numbers may be biologically plausible as multicopy extrachromosomal DNAs in bacteria can reach hundreds of copies per chromosome per cell and can reach thousands of copies per chromosome per cell in extreme cases (Maddamsetti et al. 2024). However, lower copy numbers may suffice for evolutionary persistence in practice, and further work is needed to quantify actual Obelisk-*S.s* copy numbers in SK36 and measure its evolutionary persistence in laboratory experiments.

## Discussion

Here, we confirm that Obelisk-*S.s* indeed represents a novel class of viroid-like RNA that is highly abundant in the *S. sanguinis* SK36 transcriptome. As Obelisk-*S.s* is found in transcriptomes measured by independent labs, this work agrees with prior work that intracellular Obelisk-*S.s* populations are stably maintained in SK36 (Zheludev et al. 2024b). The evolutionary forces driving Obelisk persistence are SK36 remain unknown, and basic aspects of their evolutionary dynamics, such as Obelisk mutation rates, are not understood. It is unclear whether the Obelisk-*S.s* polymorphisms reported here have any functional or adaptive significance, and the extent to which the dynamics of these polymorphisms are driven by selection is unknown. Given that Obelisk-*S.s* is likely an RNA-replicating entity, the low-frequency polymorphisms reported here could be caused by a relatively low mutation rate during RNA replication, or the action of strong purifying selection on Obelisk-*S.s* populations within *S. sanguinis* SK36. The latter hypothesis may be more likely, since viroids have some of the highest known mutation rates (Gago et al. 2009).

We can speculate on possible mechanisms driving long-term Obelisk persistence in bacterial cells. In replete growth conditions, Zheludev et al. found no growth rate difference between Obelisk-*S.s*^+^ and Obelisk-*S.s*^−^ SK36 isolates. Therefore, Obelisk-*S.s* may generally impose a negligible fitness burden on host cells. Alternatively, Obelisks may encode some beneficial function under some (unknown) conditions. Another possibility is that Obelisks could encode some post-segregational killing mechanism, much like the addictive toxin-antitoxin systems that stabilize many plasmids against stochastic loss. Such systems produce a short-lived antitoxin and a long-lived toxin; if the system is lost, the antitoxin is lost while the toxin persists, killing the cell (Jurėnas, et al. 2022).

Our mathematical model suggests that high abundance can play a transient role in stabilizing Obelisk persistence in SK36, by reducing the stochastic loss rate of Obelisks from daughter cells. The model also shows that Obelisk persistence can be stabilized indefinitely when Obelisk abundance is so high that the probability of generating a daughter cell without any Obelisks goes to zero. Therefore, the simplest hypothesis for long-term Obelisk persistence, illustrated by our mathematical model, is that Obelisks may be so abundant in SK36 that they can persist in the absence of any benefit to its host.

Finally, another possibility is that Obelisk persistence is stabilized by horizontal gene transfer (Lopatkin, et al. 2017; Maddamsetti and Lenski 2018; Wang and You 2020; Wang, et al. 2022). It is well established that *S. sanguinis* produces extracellular membrane vesicles (Choi, et al. 2018; Helliwell, et al. 2023), and it has been reported that these vesicles contain small RNAs (Choi, et al. 2018). There is also some evidence that small RNAs can be transmitted between cells, although aspects of this hypothesis remain controversial (Tsatsaronis, et al. 2018; Chen and Rechavi 2022). Therefore, we can speculate that Obelisks may undergo intercellular transfer through some unknown mechanism, perhaps involving extracellular membrane vesicles.

A key limitation of our model is that it overlooks the critical observation of spontaneous Obelisk loss in *S. sanguinis* cultures under replete conditions (Zheludev et al. 2024b). Large cellular fluctuations in intracellular Obelisk population sizes could result in the stochastic loss seen in experiments. Future experimental work could measure how Obelisk population sizes vary in single cells, to examine whether stochastic loss rates vary with environmental conditions, and to explore what molecular mechanisms lead to stochastic loss. Some possibilities include whether host regulatory mechanisms periodically purge Obelisks despite high abundance, or specific subcellular localizations of Obelisks that increase the likelihood of daughter cells that fail to inherit any Obelisks.

Fundamentally, basic aspects of Obelisk biology and function are poorly understood. Understanding how this new class of viroid-like RNA functions and evolves remains an exciting problem at the frontier of the biological sciences.

## Methods

### Obelisk-*S.s* cDNA Sequence

The following Obelisk-*S.s* cDNA sequence was reported by Zheludev et al. (2024a, b) in Supplementary_table_1_stringent_Obelisk_clustering_011724.tsv, and used for downstream sequence analysis.

 > Obelisk_000003|Obelisk-*S.s*

ACTTGGTTAGTCCAGGAACTGTAATATATTAGAAAGGAAGTAAACCAAACATGTTAGATTGGAATACCTCATCAGACATCTTCGTCGAGAAGCTTCTTCAGAGAAACTACAAGAGTCAGAGTCTGCACAGCCAACCTCGCCATCGACCCCAAGTGGATGGAATTCCTTACGAGTTTGGATACAAAGGAACGATCTATCCTCTGAATAAATCACGAAACTGTATCATCATCTTGCTGTTGATACCCATTCTAGTTCACAGTACCAGAAATGCAGCCTACTTCGAGAGTCTCGAGAAGAAAATTGTCGAGCAAGTGAAGCTAAACAGGGCTCAAGGTAAATGGCAATTAGTCAGAGAACTTCTCGGACTAAAAGGCACTTTCCTCAAGCCCCGCTGGCAACACTTTGCGAAGACAGTTTCTTCAAGAGACTTCTTCGGAAATTGGCTACCTCTGATGCTAGAAATAGAAAGGTACCTTTACAGTAAAAAGATGTATCCAGATTCATATTTATCCTGGGACGATCATTCTTCGTACCGAGTTCGCAAGAAAGTCTACCGCCGTGGTTATGACGACAAGGGTAGCCGGAGACCTGAACACAAGTGGTTCCCTGAGAATGCCTTCTCTCGAGAATTGCTTGATGAAGTTCCGGTCAAACGTGCTGTTTACAAGCCGTTCGAACTATATCATGGTTACTCTGAAAAACGAAGGCGGAGATCGTCTCTAAGTTCTCTTCTAGATTTATAGGCAACGGAAAGCCTAAGAACTTAAGGTCGAACTTCTTCTTTCAAGAATTTCCTAATTGGTAAATTCTCTCAGTAAATCAATAACTTATTTTCCTTTGGAGAATTTGTTCCCTCTGAGGAAGAAGTAAAATTAAAATTTCGGGATTTTGAGGGACGGCAATCACGTCCCCTTTTCTCTTTTCGGAGAAAAAGGGATTGGGATTACCATCCCCTTAATTCTGTAAATTTTATTTTACCTCTTTCCTCTTAGGTTCAAATCCCCCAGAAGGGAAAAGTAGTTGACTATTGAAAGTCTCTACCCTATTGGGAAAGGCTCGTCGGAAAACAGTTCTCCGAAAGTTCTCATGACATTCTGGTGTCACAAAGTCGAGATGAGAACAAAGATTCGTCTTCGG.

*RNAfold* 2.6.4 (Lorenz, et al. 2011) Was Used to Predict the Secondary Structure of Obelisk-*S.s*

### BLAST Homology Search

The NCBI BLAST web server at https://blast.ncbi.nlm.nih.gov/Blast.cgi?PROGRAM=blastn&PAGE_TYPE=BlastSearch&LINK_LOC=blasthome (Boratyn, et al. 2013) was used to compare the Obelisk-*S.s* cDNA sequence to the Nucleotide collection (nr/nt) database. The search was repeated, using the Organism field to restrict the homology search to *Streptococcus sanguinis* SK36 (taxid:388,919).

### SK36 Genome Resequencing Analysis

We manually annotated the location of the Oblin-1 in the Obelisk-*S.s* cDNA sequence and downloaded a GenBank-formatted reference sequence for Obelisk-*S.s* using Benchling.com. We downloaded a full GenBank-formatted reference sequence for the S. sanguinis SK36 genome from the NCBI RefSeq database (Haft, et al. 2024) at: https://www.ncbi.nlm.nih.gov/nuccore/NC_009009.1/ SK36 genome resequencing data were downloaded from the NCBI Short Read Archive (SRA) at: https://www.ncbi.nlm.nih.gov/sra/SRR14406732.

We then used *breseq* version 0.37.0 (Deatherage and Barrick 2014) to jointly map the Illumina sequencing reads (SRR14406732) to the SK36 reference genome and the Obelisk-*S.s* reference sequence, as follows:

breseq -j 10 -p -o../results/SK36-DNAseq-breseq-polymorphism -r../data/SK36-genome.gbk -r../data/Obelisk_000003.gbk../data/SRR14406732.fastq.gz.

This was recorded in a shell script called *map-SK36-DNAseq-data.sh*.

### SK36 Transcriptomic Data and Analysis

*Pysradb* 2.1.0 (Choudhary 2019) and *SRA Toolkit* 3.0.5 (Kodama, et al. 2012; Team 2024) were used to fetch metadata and download transcriptomic data isolated from *S. sanguinis* SK36 monocultures (Tables 1 and 2). *kallisto* (Bray, et al. 2016) was used to map the transcriptomic data for each sample in Table 1 to the SK36 reference genome and the Obelisk-*S.s* reference sequence. This was automated with a Python 3.11 script called *run-kallisto-on-SK36.py*. *breseq* version 0.37.0 (Deatherage and Barrick 2014) was used to map the transcriptomic data for each sample in Table 1 to the SK36 reference genome and the Obelisk-*S.s* reference sequence, using a shell script *map-SK36-RNAseq-data.sh*. *breseq* was run in polymorphism mode (-p option) with default parameter settings, which reports mutations at a 5% allele frequency threshold.

### Mathematical Model

We built a mathematical model to examine how Obelisk copy number affects Obelisk evolutionary stability. A diagram of the model is shown in Fig. 1B. This model involves two subpopulations of bacteria. The first carries an intracellular population of Obelisks (Type 1) and the second is an Obelisk-free subpopulation (Type 2). We are interested in the dynamics of these two populations due to selection (growth) and mutation (stochastic loss of intracellular Obelisks during cell division).

An interactive Pluto computational notebook of the model, called *obelisk-abundance-model.jl*, is available at: https://github.com/rohanmaddamsetti/obelisk-SK36/. This notebook can be run by installing and running Pluto.jl within Julia 1.11 + (see instructions at: https://plutojl.org/) and then opening the notebook using the Pluto web browser interface. Unless otherwise stated, the simulation results shown in Fig. 1 use the following default parameter settings (units of Obelisk copies per cell): Obelisk copy number *N* = 10, Obelisk capacity N* = 1000. The choice of *N* = 10 and N* = 1000 is arbitrary, as the results in Fig. 1 do not change with different parameter settings. Interested readers can try this for themselves using the interactive computational notebook.

#### Model Assumptions

The population is modeled as a vector of two states, **x**, where *x*_1_ represents a subpopulation containing intracellular Obelisks, and *x*_2_ represents a subpopulation that has lost the Obelisks.

$$ {\varvec{x}} = \left[ {\begin{array}{*{20}c} {x_{1} } \\ {x_{2} } \\ \end{array} } \right] $$

The total population size *X*(*t*) = *x*_1_(*t*) + *x*_2_(*t*).

*Selection dynamics.* We assume that subpopulation *x*_1_ grows at rate *r*_1_, and subpopulation *x*_2_ grows at rate *r*_2_. We use the term "fitness" synonymously with these growth rates. We assume that *r*_1_ = *r*_2_ – α*N*, where *N* is the Obelisk copy number per cell. This assumption means that Obelisks have no fitness benefit and confer a small linear fitness cost α per copy. We define N* as the Obelisk capacity; that is, the Obelisk copy number at which *r*_1_ = 0 (Fig. 1D). So,

$$ \alpha = \frac{{r_{2} }}{{N^{*} }} $$

$$ r_{1} = r_{2} - \frac{{r_{2} N}}{{N^{*} }} $$

$$ r_{1} = r_{2} \left( {1 - \frac{N}{{N^{*} }}} \right) $$

In our model, Obelisk copy number *N* and Obelisk capacity N* are free parameters, under the assumption that 0 ≤ *N* < N*.

*Mutation dynamics*. We assume that *x*_1_ cells generate daughter cells at rate *r*_1_ and that those daughter cells fail to inherit any Obelisks with probability *µ*. We model *µ* as a geometrically decreasing function of Obelisk copy number *N*, based on the following argument:

Suppose a cell with Obelisks divides. The per-capita rate of generating a cell without Obelisks is the probability that one daughter cell gets no Obelisks and the other gets all of its parent’s Obelisks (Fig. 1C). We assume partitioning is purely stochastic. So, the probability that a daughter doesn’t get an Obelisks is:

$$ \mu = \left( \frac{1}{2} \right)^{N} $$

Since the per-capita growth rate for Type 1 cells is *r*_1_, the per-capita rate of generating Obelisk-free daughter cells is *r*_1_*µ*, and the per-capita rate of generating daughter cells with Obelisks is *r*_1_ (1 – *µ*).

*Full dynamics.* The selection and mutation dynamics are combined into a matrix **A**:

$$ {\varvec{A}} = \left[ {\begin{array}{*{20}c} {r_{1} \left( {1 - \mu } \right)} & 0 \\ {r_{1} \mu } & {r_{2} } \\ \end{array} } \right] $$

So, the full dynamics are modeled by the following matrix system of ODEs (Fig. 1B):

$$ \frac{{d{\varvec{x}}}}{dt} = {\varvec{Ax}}\left( t \right) $$

$$ \frac{{d{\varvec{x}}}}{dt} = \left[ {\begin{array}{*{20}c} {r_{1} \left( {1 - \mu } \right)} & 0 \\ {r_{1} \mu } & {r_{2} } \\ \end{array} } \right]\left[ {\begin{array}{*{20}c} {x_{1} \left( t \right)} \\ {x_{2} \left( t \right)} \\ \end{array} } \right] $$

*Analytical solution for response time*. Consider the initial condition $${\varvec{x}}(0)= \left[\begin{array}{c}1\\ 0\end{array}\right]$$; that is, *x*_1_(0) = 1 and *x*_2_(0) = 0. Since we assume that *r*_1_ < *r*_2_, eventually *x*_1_ ≤ *x*_2_. How long does it take for *x*_2_ = *x*_1_? This is a natural measure of the transient stability of the *x*_1_ state. We have a linear system of ODEs that can solved analytically:

$$ \frac{{d{\varvec{x}}}}{dt} = {\varvec{Ax}}\left( t \right) $$

$$ \frac{{d{\varvec{x}}}}{dt} = \left[ {\begin{array}{*{20}c} {r_{1} \left( {1 - \mu } \right)} & 0 \\ {r_{1} \mu } & {r_{2} } \\ \end{array} } \right]\left[ {\begin{array}{*{20}c} {x_{1} \left( t \right)} \\ {x_{2} \left( t \right)} \\ \end{array} } \right] $$

The eigenvalues of **A** are the diagonal entries *r*_1_(1–*µ*) and *r*_2_, as these are the roots of the characteristic polynomial of **A**. Solving for the corresponding eigenvectors, we find the following general solution:

$$ {\varvec{x}}\left( t \right) = c_{1} \left[ {\begin{array}{*{20}c} { - 1} \\ {\frac{{r_{1} \mu }}{{r_{2} - r_{1} \left( {1 - \mu } \right)}}} \\ \end{array} } \right]e^{{r_{1} \left( {1 - \mu } \right)t}} + c_{2} \left[ {\begin{array}{*{20}c} 0 \\ 1 \\ \end{array} } \right]e^{{r_{2} t}} $$

Using the initial condition $${\varvec{x}}(0)= \left[\begin{array}{c}1\\ 0\end{array}\right]$$, we find that:

$$ c_{1} = - 1 $$

$$ c_{2} = \frac{{r_{1} \mu }}{{r_{2} - r_{1} \left( {1 - \mu } \right)}} $$

So the particular solution is:

$$ {\varvec{x}}\left( t \right) = \left[ {\begin{array}{*{20}c} 1 \\ {\frac{{ - r_{1} \mu }}{{r_{2} - r_{1} \left( {1 - \mu } \right)}}} \\ \end{array} } \right]e^{{r_{1} \left( {1 - \mu } \right)t}} + \left[ {\begin{array}{*{20}c} 0 \\ {\frac{{r_{1} \mu }}{{r_{2} - r_{1} \left( {1 - \mu } \right)}}} \\ \end{array} } \right]e^{{r_{2} t}} $$

Now, we can solve for the response time t* where *x*_1_(t*) = *x*_2_(t*). Some algebra yields:

$$ t^{*} = \frac{{{\text{log}}\left( {1 + \frac{{r_{2} - r_{1} \left( {1 - \mu } \right)}}{{r_{1} \mu }}} \right)}}{{r_{2} - r_{1} \left( {1 - \mu } \right)}} $$

where log() is the natural logarithm (base *e*). Without loss of generality, we can let *r*_2_ = 1, so that ${r}_{1}=\left(1- \frac{N}{{N}^{*}}\right)$ and $\upmu ={\left(\frac{1}{2}\right)}^{N}$ and examine how t* changes as we change Obelisk copy number *N* and Obelisk capacity N* (which defines the burden or fitness cost of an intracellular Obelisk population). This result is shown in Fig. 1H.

## Supplementary Information

Below is the link to the electronic supplementary material.

Supplementary file1 (xlsx 2482 kb)

## Data Availability

All data analyzed in this work is publicly available. NCBI accessions are provided in the Tables and Methods. All data analysis code is available at: https://github.com/rohanmaddamsetti/obelisk-SK36.
